# Macrophage and *Galleria mellonella *infection models reflect the virulence of naturally occurring isolates of *B. pseudomallei*, *B. thailandensis *and *B. oklahomensis*

**DOI:** 10.1186/1471-2180-11-11

**Published:** 2011-01-17

**Authors:** Matthew E Wand, Claudia M Müller, Richard W Titball, Stephen L Michell

**Affiliations:** 1 Biosciences, College of Life and Environmental Sciences, Geoffrey Pope Building, University of Exeter, Stocker Road, Exeter, Devon, EX4 4QD, UK; 2 Health Protection Agency, Porton Down, Salisbury SP4 0JG, UK

## Abstract

**Background:**

*Burkholderia pseudomallei *is the causative agent of melioidosis, a tropical disease of humans with a variable and often fatal outcome. In murine models of infection, different strains exhibit varying degrees of virulence. In contrast, two related species, *B. thailandensis *and *B. oklahomensis*, are highly attenuated in mice. Our aim was to determine whether virulence in mice is reflected in macrophage or wax moth larvae (*Galleria mellonella*) infection models.

**Results:**

*B. pseudomallei *strains 576 and K96243, which have low median lethal dose (MLD) values in mice, were able to replicate and induce cellular damage in macrophages and caused rapid death of *G. mellonella*. In contrast, *B. pseudomallei *strain 708a, which is attenuated in mice, showed reduced replication in macrophages, negligible cellular damage and was avirulent in *G. mellonella *larvae. *B. thailandensis *isolates were less virulent than *B. pseudomallei *in all of the models tested. However, we did record strain dependent differences. *B. oklahomensis *isolates were the least virulent isolates. They showed minimal ability to replicate in macrophages, were unable to evoke actin-based motility or to form multinucleated giant cells and were markedly attenuated in *G. mellonella *compared to *B. thailandensis*.

**Conclusions:**

We have shown that the alternative infection models tested here, namely macrophages and *Galleria mellonella*, are able to distinguish between strains of *B. pseudomallei*, *B. thailandensis *and *B. oklahomensis *and that these differences reflect the observed virulence in murine infection models. Our results indicate that *B. oklahomensis *is the least pathogenic of the species investigated. They also show a correlation between isolates of *B. thailandensis *associated with human infection and virulence in macrophage and *Galleria *infection models.

## Background

*Burkholderia pseudomallei *is a facultative intracellular pathogen responsible for melioidosis, an infectious disease of humans prevalent in Southeast Asia and Northern Australia [1]. Infections in humans may result in a wide range of clinical symptoms and manifestations [2,3] and in some individuals the bacterium is able to persist with symptoms not shown until several years after exposure [4].

*B. pseudomallei *has been shown to have a broad host range with disease reported in animals ranging from kangaroos to dolphins [5,6]. However, in the laboratory, the mouse is the most commonly used infection model [7]. Different strains of *B. pseudomallei *vary markedly in their virulence in murine models of disease. When given by the intraperitoneal (i.p) route, the most virulent isolates have an infectious dose of less than 50 colony forming units (cfu), whereas in the least virulent isolates the infectious dose is over 5,000 cfu [7]. It is not clear whether these differences in virulence in mice are associated with the various clinical outcomes observed in humans.

Whilst murine models of infection are valuable for understanding mechanisms of virulence, the behaviour of *B. pseudomallei *in cell culture systems has been used to characterise the intracellular lifestyle of the bacterium. *B. pseudomallei *has been shown to be taken up by professional phagocytes including mouse macrophage-like cell lines such as J774 and RAW264 [8,9] and non-phagocytic cells including HeLa and A549 cells [8].

More recently, other members of the *Burkholderia *genus including *B. thailandensis *and *B. oklahomensis *have been described as being closely related to *B. pseudomallei *[10,11]. Indeed, until recently, *B. thailandensis *isolates were classified as *B. pseudomallei *[10]. There is extensive chromosomal synteny between *B. thailandensis *and *B. pseudomallei*, although some virulence-associated genes which are present in *B. pseudomallei *are absent in *B. thailandensis *[12]. Both *B. pseudomallei *and *B. thailandensis *are able to invade and grow in a range of phagocytic and non-phagocytic cells, forming plaques or multinucleated giant cells [13,14]. However, there is also evidence that the behaviour of *B. pseudomallei *and *B. thailandensis *differs in different cell lines. In A549 and human dendritic cells, *B. pseudomallei *has been shown to be more invasive than *B. thailandensis*, but there were no reported differences in the growth rate within cells. In contrast, in human macrophages, differences in intracellular growth rates have been reported [14]. Collectively, these findings have suggested that *B. thailandensis *could be used as a model to study certain aspects of the intracellular lifestyle of *B. pseudomallei *in cell culture systems [15]. The behaviour of *B. oklahomensis *in cell culture models is not known.

The value of whole animal or plant infection models, which use *B. thailandensis *or *B. oklahomensis *in place of *B. pseudomallei*, is much less clear. Isolates of *B. thailandensis *and *B. oklahomensis *that have been tested are considered to be highly attenuated or avirulent in BALB/c mice, with lethal doses for most isolates in excess of 10^7 ^cfu by the i.p. route [16]. However, using intranasal challenge models, doses of greater than 10^4 ^cfu of *B. thailandensis *are reportedly able to kill mice and replicate *B. pseudomallei *disease phenotypes, although even in this model it is clear that *B. thailandensis *is much less virulent than *B. pseudomallei *[7].

There has been significant interest in the development of alternative infection models which avoid the use of mammals but also reflect the differences in virulence of species and isolates seen in mice. The *Caenorhabditis elegans *[17] or tomato plant [18] infection models were not able to distinguish between *B. pseudomallei *and *B. thailandensis*, and in *C. elegans, B. thailandensis *was the most virulent [17]. *Galleria mellonella *(wax moth) larvae have previously been reported as susceptible to infection with *B. pseudomallei*, and a single *B. thailandensis *strain tested was reportedly less virulent [19]. This finding suggests that *G. mellonella *larvae may be a suitable host species for discerning differences in virulence.

Our aim was to determine whether differences in the virulence of *B. pseudomallei*, *B. thailandensis *and *B. oklahomensis *isolates could be reliably determined in macrophage and *G. mellonella *larvae infection models.

## Results

### *B. pseudomallei*, *B. thailandensis *or *B. oklahomensis *are internalised with similar efficiencies into J774A.1 macrophages

For this study we have selected a range of *B. pseudomallei*, *B. thailandensis *or *B. oklahomensis *isolates of known ancestry. The properties of these isolates and their virulence in murine models of disease is summarised in Table 1. The intracellular replication profiles for each isolate were initially determined in a cell culture model using murine macrophages. This was performed using a modified intracellular replication assay where 250 μg/ml kanamycin was used to kill extracellular bacteria, as validated below. Initially, the minimum inhibitory concentration (MIC) of kanamycin for each strain was determined and found to be 16-128 μg/ml (Table 1). All of the strains tested were unable to grow in the presence of 250 μg/ml kanamycin in broth. Similarly, supernatants of J774A.1 cell cultures containing 250 μg/ml kanamycin and infected with any of the strains did not contain viable bacteria when samples were plated onto agar. To test for harmful effects of kanamycin on eukaryotic cell lines, cell toxicity assays (LDH assays) were carried out on culture supernatants from uninfected J774A.1 cells that had been cultured in the presence of 250 μg/ml kanamycin. There was no significant difference between the LDH levels of these culture supernatants compared to control supernatants from J774A.1 cells cultured in the absence of kanamycin (data not shown).

**Table 1:** *Burkholderia *isolates used in this study.

Isolate	Description and reference	MIC (μg/ml kanamycin)	Virulence in mice by i.p. route
*B. pseudomallei*			
K96243	Clinical isolate from Thailand, sequenced strain [26]	128	MLD = 262 (i.p.) [7]
576	Clinical isolate from Thailand [28]	128	MLD = 80 (i.p.) [7]
708a	Gentamicin-sensitive isolate from Thailand [9]	16	MLD = 2.3 × 10^3 ^(i.p.) [7]
*B. thailandensis*			
E264	Environmental isolate, sequenced strain [10,37]	128	1/10 survivors at 10^7 ^cfu [16]
Phuket 4W-1	Water isolate from Thailand [38]	128	2/10 survivors at 10^7 ^cfu [16]
CDC3015869	Clinical isolate from Texas; abbreviated as CDC301 [39]	128	8/10 survivors at 10^7 ^cfu [16]
CDC2721121	Clinical isolate from Louisiana; abbreviated as CDC272 [39]	128	10/10 survivors at 10^7 ^cfu [16]
*B. oklahomensis*			
C6786	Clinical isolate from Oklahoma [40]	128	10/10 survivors at 10^7 ^cfu [16]
E0147	Clinical isolate from Georgia [41]	128	10/10 survivors at 10^7 ^cfu [16]

Description of the *Burkholderia *strains used in this study, their susceptibility to kanamycin as described by the minimum inhibitory concentration (MIC) and a summary of published data on virulence of these isolates in mice described as the median lethal dose (MLD) in colony forming units or as number of survivors.

**Figure 1:**
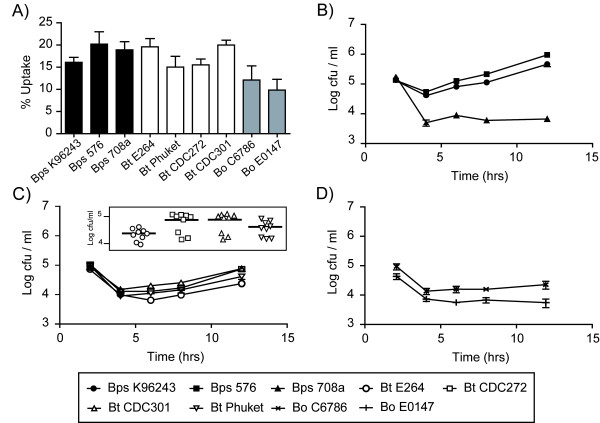
**Bacterial uptake, intracellular survival and replication of *Burkholderia *strains in mouse macrophages**. J774A.1 macrophages were exposed to *Burkholderia *strains at an MOI of 10 and the mean numbers of intracellular bacteria were determined at 2, 4, 6, 8 and 12 hrs post infection. (A) Uptake of bacteria by macrophages as determined by bacterial counts 2 hrs post infection relative to the input numbers. (B-D) Intracellular survival and replication of *B. pseudomallei *(Bps; panel B), *B. thailandensis *(Bt; panel C) and *B. oklahomensis *(Bo; panel D) in J774A.1 macrophage cells. Error bars represent the standard error of the mean. All infections were performed as three independent experiments, each with three technical replicates. The insert in panel C represents individual bacterial counts and the mean value at 12 hrs post infection with different *B. thailandensis *strains.

The first parameter that was assessed in the macrophage model was internalisation efficiencies of the *Burkholderia *strains. Bacteria released from J774A.1 macrophages lysed 2 hrs post infection were enumerated on agar plates and compared to the input number. There was no significant difference between the degree of internalisation of *B. pseudomallei*, *B. thailandensis *or *B. oklahomensis *into murine macrophages (Figure 1A).

### High virulence isolates of *B. pseudomallei *grow more rapidly in J774A.1 macrophages than low virulence isolates, *B. thailandensis *or *B. oklahomensis*

Next, intracellular replication was measured at 2, 4, 6, 8 and 12 hrs post infection. There was a significant difference between the numbers of intracellular *B. pseudomallei *strains 576 and K96243 at 12 hrs post infection (P = 0.002; Figure 1B) and both were significantly higher than numbers of intracellular *B. pseudomallei *strain 708a and any of the *B. thailandensis *or *B. oklahomensis *strains tested (P < 0.002, both). Bacterial numbers were over 10-fold lower with any of the *B. thailandensis *or *B. oklahomensis *strains tested (compare Figure 1B to Figure 1C&1D). To test whether the low numbers of intracellular bacteria observed with *B. pseudomallei *708a, which is more sensitive to kanamycin, was a consequence of low levels of antibiotic crossing the eukaryotic cell membrane, J774A.1 cells were infected with *B. thailandensis *DW503 (an *amrAB-oprA *efflux pump mutant and therefore highly sensitive to kanamycin) and intracellular bacterial numbers were compared to its parental strain E264. The numbers of bacteria isolated at each time point were not significantly different between strains E264 and DW503 (data not shown).

Our results also showed variance between the patterns of growth in macrophages of different isolates of *B. thailandensis*. The *B. thailandensis *strains previously isolated from cases of human disease, CDC272 and CDC301, showed increased numbers at 12 hrs post infection relative to *B. thailandensis *E264 (P < 0.004, both; see insert in Figure 1C) and the two *B. oklahomensis *strains C6786 and E0147 (P < 0.009, both), but not *B. thailandensis *strain Phuket (P > 0.05). To show that these differences in bacterial numbers were due to differences in intracellular replication and survival rather than a difference in bacterial fitness, growth rates of bacteria in antibiotic free media were compared. There was no significant difference between any of the strains tested (data not shown).

### Cellular damage assessed by LDH release correlates with virulence

**Figure 2:**
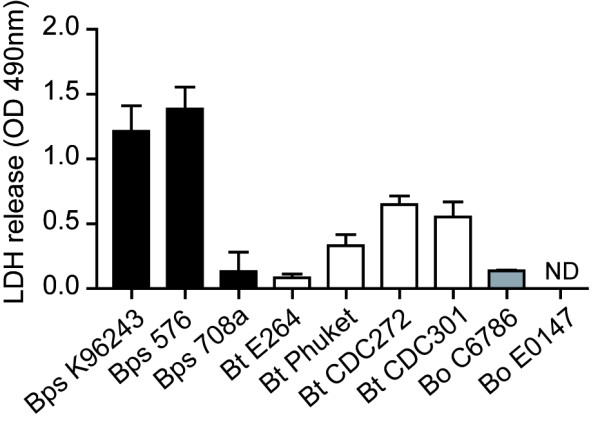
**Cellular damage in macrophages caused by invasion of *Burkholderia *as measured by LDH release**. J774A.1 macrophages were infected with *Burkholderia *strains at an MOI of 10 as already described and culture supernatants were analysed at 24 hrs post infection. The release of lactate dehydrogenase (LDH) from damaged or lysed cells was measured as described in the method section using a calorimetrical assay. Supernatants from uninfected macrophages were used to obtain a background OD 490 nm value, which was subtracted from the sample measurements. The error bars represent the standard error of the mean derived from three independent experiments, each performed in three technical replicates. ND = not detected.

The ability to cause cellular damage is often used as an indicator of bacterial virulence. Cellular damage can be measured by the release of lactate dehydrogenase (LDH) from dead or dying cells. J774A.1 macrophages were challenged with bacteria and LDH levels in supernatants were measured at 12 and 24 hrs post infection. At 12 hrs, LDH levels were relatively low and there was no significant difference in the levels of LDH released from cells infected with any of the bacteria tested (data not shown). However, at 24 hrs, the levels of LDH in the supernatants of cells infected with *B. pseudomallei *strains 576 or K96243 was higher than the LDH levels in cell supernatants infected with other *Burkholderia *strains (P < 0.03, both; Figure 2). Supernatants from cells infected with *B. thailandensis *strains CDC272, CDC301 and Phuket contained elevated levels of LDH relative to uninfected controls, but supernatants from cells infected with *B. pseudomallei *708a, *B. thailandensis *E264 or either *B. oklahomensis *strain contained negligible levels of LDH.

### *B. thailandensis *but not *B. oklahomensis *is able to cause multinucleated giant cell formation

**Figure 3:**
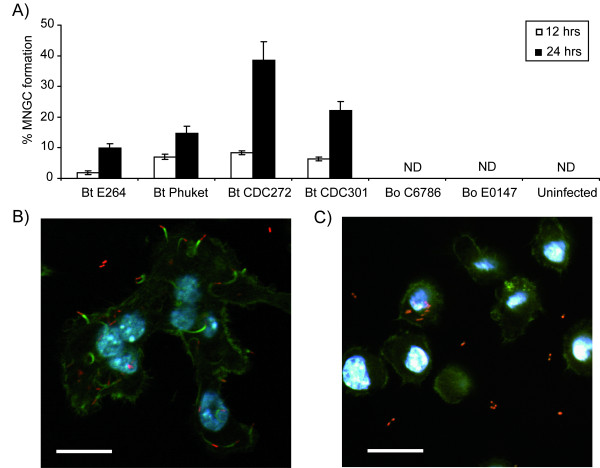
**MNGC formation and intracellular behaviour of *Burkholderia *strains in macrophages**. J774A.1 macrophages were infected with *Burkholderia *strains at an MOI of 10 as already described. (A) Multinucleated giant cell (MNGC) formation was assessed at 12 hrs and 24 hrs post infection. Cells were stained with Giemsa and the percentage of MNGCs was calculated relative to normal macrophages per field of view. MNGCs were defined as cells containing 3 or more nuclei. The error bars represent the standard error of the mean derived from at least 10 fields of view. ND = not detected. (B-C) Representative confocal micrographs of cells at 8 hrs post infection with *B. thailandensis *strain E264 (B) and *B. oklahomensis *strain C6786 (C). In both panels, bacteria appear red due to expression of RFP from the modified broad-host-range vector pBHR4-groS-RFP. Filamentous actin was stained green with FITC-phalloidin conjugate and nuclei were stained with DAPI. Scale bars represent 20 μm.

*B. pseudomallei *has previously been shown to form multinucleated giant cells (MNGCs) upon invasion of macrophages [20]. Here, *B. thailandensis *and *B. oklahomensis *strains were tested for their ability to form MNGCs after infecting J774A.1 macrophages. A cell was considered to be a MNGC if there were 3 or more nuclei present. *B. thailandensis *was able to induce MNGC formation in a strain dependent manner. *B. thailandensis *strains CDC272 and CDC301 were most effective at causing MNGC formation (Figure 3A). In contrast, *B. thailandensis *strain E264 was poor at causing the formation of MNGCs and the *B. oklahomensis *strains tested did not appear to induce MNGC formation beyond uninfected background levels. A representative confocal microscopy image of a MNGC formed by *B. thailandensis *is shown in Figure 3B.

### *B. thailandensis *but not *B. oklahomensis *exhibits actin-based motility in J774A.1 macrophages

Actin-based motility on infection of eukaryotic cells has previously been demonstrated for *B. pseudomallei *[20,21] and *B. thailandensis *strain E30 [22]. To determine whether other *B. thailandensis *strains and *B. oklahomensis *are also able to migrate using actin-based motility, J774A.1 macrophages were infected with strains that expressed red fluorescent protein from plasmid pBHR4-groS-RFP. In preliminary studies, we showed that the presence of the plasmid did not affect the growth of the bacteria in LB broth or inside macrophages, and the plasmid was stably maintained for the course of the intracellular replication assay. At different time points post infection, macrophages were stained with Phalloidin conjugated to FITC and analysed by confocal microscopy. Both *B. thailandensis *and *B. oklahomensis *were visualised in the cells. Actin tails were visible and associated with *B. thailandensis *(Figure 3B) but were not visible in *B. oklahomensis *infected cells (Figure 3C).

### Infection of *Galleria mellonella *larvae with *Burkholderia*

**Figure 4:**
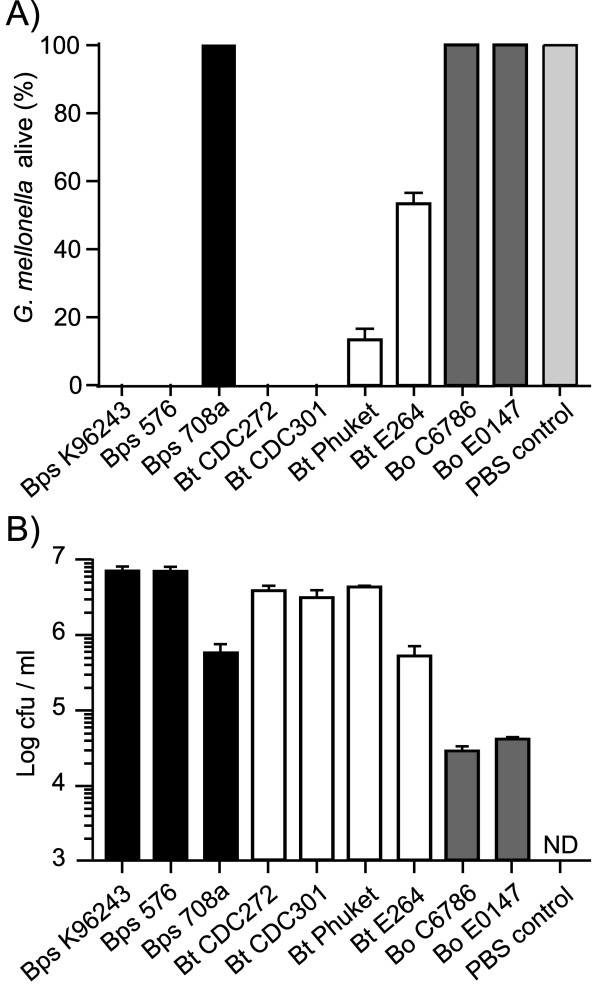
**Virulence and intracellular survival of *Burkholderia *strains in *Galleria mellonella *larvae**. Groups of 10 insect larvae were challenged with 100 cfu of different strains of *Burkholderia *as described in the method section. A) Percentage of surviving larvae at 24 hrs post infection. B) Number of bacteria present inside the haemocoel at 20 hrs post infection (calculated as cfu/ml). In both panels, results are shown as means and standard error of the mean of three independent experiments. *B. pseudomallei *= black bars; *B. thailandensis *= white bars and *B. oklahomensis *strains = grey bars. ND = not detected.

*Galleria mellonella *(wax moth) larvae were challenged with approximately 100 cfu of *B. pseudomallei*, *B. thailandensis *or *B. oklahomensis *and survival was recorded at 24 hrs post-challenge. *B. pseudomallei *strains 576 or K96243 caused 100% mortality, but no deaths were observed after challenge with *B. pseudomallei *708a (Figure 4A). Challenge with *B. oklahomensis *strains C6786 or E0147 also did not result in death of the larvae at 24 hrs post infection. The *B. thailandensis *strains showed different degrees of virulence in this model. 100% mortality was recorded after challenge with *B. thailandensis *CDC272 or CDC301. Challenge with *B. thailandensis *Phuket or E264 resulted in mortality of approximately 80% and 50% of larvae, respectively (Figure 4A). At 20 hrs post challenge, just prior to the onset of paralysis and death, larvae were sacrificed and the number of bacteria in the haemocoel was enumerated. For all of the strains tested, the bacterial numbers at 20 hrs post infection were higher than the input number (Figure 4B). Similar to the cell culture model, *B. pseudomallei *strains 576 and K96243 and *B. thailandensis *strains CDC272, CDC301 and Phuket showed increased bacterial numbers relative to *B. pseudomallei *708a, *B. thailandensis *E264 and both *B. oklahomensis *strains. To show that live bacteria are needed for killing of *G. mellonella*, *B. thailandensis *CDC272 or CDC301 were inactivated by heating to 80°C for 1 hour and then injected into *G. mellonella *larvae at the same concentration as live bacteria. After 24 hrs, all larvae infected with heat killed bacteria were still alive, whereas those infected with live bacteria had all died (data not shown).

At higher challenge doses of 10,000 cfu bacteria, all of the strains caused 100% mortality of the cohort of larvae at 24 hrs post injection, except *B. pseudomallei *708a, *B. thailandensis *DW503 and *B. oklahomensis *E0147. At lower inocula of 10 cfu bacteria, all of the *B. pseudomallei *strains were able to kill *G. mellonella *by 72 hrs post challenge, but no dead larvae were recorded up to 5 days after challenge with *B. thailandensis *or *B. oklahomensis*.

## Discussion

In this study, we set out to identify inexpensive alternative infection models that would reflect the virulence of *B. pseudomallei*, *B. thailandensis *or *B. oklahomensis *in mice and the association of these isolates with human disease. We have chosen *B. pseudomallei *isolates with different degree of virulence in mice, with strain 576 representing one of the most virulent isolates tested to date, and 708a one of the least [7]. *B. thailandensis *and *B. oklahomensis *are not normally considered to be human pathogens. However, occasional cases of disease do occur. We have included clinical isolates of *B. thailandensis *in our study alongside *B. thailandensis *isolates that have not been associated with disease (E264 and Phuket), as well as clinical isolates of *B. oklahomensis*.

In general, our results confirm that cell culture or *Galleria *infection models can be used to discriminate *B. pseudomallei*, *B. thailandensis *and *B. oklahomensis *isolates and these results parallel those found in mice. With the exception of strain 708a and compared with *B. thailandensis *and *B. oklahomensis *isolates, the *B. pseudomallei *isolates we tested grew more rapidly in macrophages, caused a greater degree of cellular damage and caused greater mortality of *G. mellonella *larvae. The *B. oklahomensis *isolates we tested were the least virulent in all of these models. Our finding that we are able to distinguish between *B. pseudomallei *and *B. thailandensis *isolates on the basis of their virulence in *G. mellonella *indicates that this model has a greater utility for the identification of virulence factors than the *C. elegans *[17] or tomato plant [18] infection models.

There were some important differences in the relative virulence of isolates within each species in our models which are not reflected in mouse virulence data. In our macrophage and *G. mellonella *models, *B. pseudomallei *708a was highly attenuated, to a level similar to that of the least virulent *B. thailandensis *isolates and both of the *B. oklahomensis *isolates. However, *B. pseudomallei *708a is reported to be significantly more virulent than any *B. thailandensis *and *B. oklahomensis *isolates in mice [7,16,23]. *B. pseudomallei *708a is a naturally occurring gentamicin sensitive isolate that, when compared to *B. pseudomallei *K96243, contains a 131-kb deletion within chromosome I [23]. This deletion removes the *amrAB-oprA *operon providing aminoglycoside resistance, which explains the low MIC of kanamycin for this strain (Table 1). The deletion also results in loss of genes coding for the anaerobic arginine deiminase pathway, clusters encoding cobalamin and malleobactin iron uptake systems, and a putative type-1 fimbrial gene cluster [23]. Transcriptome data obtained from *B. pseudomallei *K96243 at day three after intranasal infection of BALB/c mice showed that genes involved in iron acquisition, including the malleobactin operon, were induced *in vivo *compared to bacteria grown *in vitro *in LB broth (C. Müller, unpublished data). The same genes are also upregulated under low iron conditions [24,25], which suggests that *B. pseudomallei *encounters iron limited conditions in the mouse model of infection. The absence of these siderophore systems in strain 708a might also partly explain the observed intracellular replication defect in macrophages (Figure 1B). Overall, and bearing in mind the genome plasticity of *B. pseudomallei *[26], we cannot be certain that the *B. pseudomallei *708a isolate we have used in our study was genetically similar to the isolate previously tested in mice. It would therefore be valuable to re-test the *B. pseudomallei *708a isolate we have used for virulence in mice.

We also identified differences in the virulence of *B. thailandensis *isolates, which were consistent between our macrophage growth, macrophage killing and *G. mellonella *models, but not with previously reported data on virulence in mice or hamsters. In our models, CDC301 and CDC272 were the most virulent isolates, whereas CDC301, E264 and Phuket were most virulent in mouse and hamster infection models [16]. A recent study revealed that both CDC strains belong to the same sequence type and are part of a distinct phylogenetic subgroup of *B. thailandensis *isolates that is separate from strains isolated in Thailand [27]. Moreover, a strain belonging to the same subgroup as the CDC strains was shown to have an increased ability to replicate in macrophages compared to strain E264, in agreement with our data presented in Figure 1C. The same study [27] revealed that CDC301 encodes a gene cluster with 94% nucleotide similarity to the capsular polysaccharide biosynthesis cluster of *B. pseudomallei*, which has been shown to play a role in virulence in mice and in hamsters [28,29]. However, our observation that strain CDC272, which does not express the Bp-like capsular polysaccharide, is as virulent as strain CDC301 in the *G. mellonella *model suggests that the capsular polysaccharide cluster is not required for virulence in insects. Overall, our results show that human clinical isolates of *B. thailandensis *are more virulent in macrophage and *G. mellonella *models, and the proposal that clinical *B. thailandensis *isolates from the USA are less virulent than SE Asian isolates [16] is not borne out by our data. At this time it is not clear whether murine, hamster, macrophage or *G. mellonella *models reflect virulence of these isolates in humans.

Our finding that the *B. oklahomensis *isolates have low virulence in macrophage or *G. mellonella *models is consistent with the report that these isolates exhibit low virulence in murine or hamster models [16]. Our work also identifies some possible reasons for this. Although we were able to visualise RFP-labelled *B. oklahomensis *cells in macrophages, we did not observe actin tail formation, suggesting that the bacteria would not be able to spread from cell to cell in the same way as *B. thailandensis *or *B. pseudomallei *[20-22]. MNGCs also failed to form in cells infected with *B. oklahomensis*, though this may simply reflect the inability of the bacteria to grow in J774A.1 macrophages. Actin-based motility in *B. pseudomallei *is dependent on BimA, which nucleates actin polymerisation [30]. Our analysis of the *B. oklahomensis *shotgun genome sequences [Genebank accession numbers NZ_ABBG01000000 and NZ_ABBF01000000] indicated the presence of a BimA-like protein with 46% overall identity to its orthologue in *B. thailandensis *E264 (BTH_II0875), and 40% identity to the *B. pseudomallei *K96243 protein (BPSS1492). The last 160 amino acids of the BimA orthologues were found to be highly conserved between all species, whereas the N-terminus exhibited considerable variation. The *B. oklahomensis *BimA proteins contain *B. mallei*-like signal peptide and proline-rich domains and a *B. thailandensis*-like central acid domain, but seem to lack a WASP homology domain-2 [22]. Therefore, it is not clear if *B. oklahomensis *BimA is functional in promoting actin polymerisation. Intracellular replication and endosomal escape of *B. pseudomallei *depends on the type III secretion system TTSS-3 [21], which is also present in *B. thailandensis *[31]. Our analysis of the *B. oklahomensis *genomes revealed the presence of a TTSS3 gene cluster, with homologies of the encoded proteins ranging from 45% to 98% compared to the *B. pseudomallei *K96243 orthologues. However, two genes, BPSS1553 (*bprP*) and BPSS1554 (*bprQ*), which have recently been identified as ToxR and ToxS-like regulators encoded directly downstream of the TTSS in *B. pseudomallei *[32], are also found in *B. thailandensis *but are absent in the *B. oklahomensis *strains. BprP activates the expression of TTSS genes, and a *bprP *mutant in *B. pseudomallei *does not secrete TTSS effector proteins and is unable to kill macrophages [32]. The absence of this activator in *B. oklahomensis *might therefore explain the low virulence of this species.

In this study we have not tested *Burkholderia mallei*, another species closely related to *B. pseudomallei*, for virulence in cell culture or *Galleria *models. It is known that *B. mallei *is able to infect and grow in macrophages [33] and to kill *G. mellonella *larvae [19]. However, the pathogenesis of *B. mallei *infection in *G. mellonella *may be quite different from the pathogenesis of *B. thailandensis *or *B. pseudomallei *infection we report here. Whereas we recorded larval death by 24 hrs post challenge with typical *B. pseudomallei *isolates, larval deaths occurred over the period 24 - 144 hrs post challenge with *B. mallei *[19]. This might be explained by the restricted host range of the obligate intracellular bacterium *B. mallei *compared to *B. pseudomallei *with its much more versatile genome [34].

## Conclusions

Our findings indicate that murine macrophage cell culture or *Galleria *infection models can be used to discriminate *B. pseudomallei*, *B. thailandensis *and *B. oklahomensis *isolates on the basis of their virulence. In general, our results support the proposal that the virulence of isolates in these models reflects virulence in murine models of disease. However, some important exceptions merit further investigation which is not within the scope of this study. Our finding that virulence of three *B. pseudomallei *isolates with high, intermediate and low virulence in mice is reflected in their virulence in cell culture or *Galleria *infection models indicates the potential value of these models for the identification of virulence-associated genes. Our findings support the proposal that *B. oklahomensis *isolates are of low virulence and indicate that these isolates are defective in growth in macrophages and in actin-based motility within cells.

## Methods

### Bacterial strains and growth conditions

The *Burkholderia *strains used in this study are summarised in Table 1. All strains were grown in LB broth with aeration or on LB agar plates at 37°C unless otherwise stated. When appropriate, antibiotics (Sigma-Aldrich) were used at the following concentrations, unless otherwise stated: kanamycin, 50 μg/ml; chloramphenicol, 25 μg/ml; and gentamicin, 50 μg/ml.

### Cell lines

J774A.1 mouse macrophage cell lines were maintained at 37°C under 5% CO_2 _atmosphere in DMEM (Hyclone) supplemented with 10% fetal bovine serum (Hyclone), 1% L-glutamine (250 mM) (Hyclone) and 1% Penicillin/Streptomycin solution (Hyclone).

### Construction of fluorescent reporter plasmids

The construction of the red fluorescent reporter plasmid used for confocal-laser scanning microscopy was performed in several steps. Firstly, a multiple cloning site (MCS) was introduced into the commercially available, mobilisable broad host range vector pBHR1 (MoBiTec; Km^R^, Cm^R^) by excising a 1.6-kb *BstB*I fragment containing a MCS within the *lacZ *gene and the chloramphenicol resistance gene from plasmid pBBR-MCS1 [35] and cloning it into the 4.5-kb fragment that resulted from cutting pBHR1 with *BstB*I. The resulting plasmids pBHR-MCS1 and pBHR-MCS2 contained the *lacZ-cat *insert in different orientations; only pBHR-MCS1 was used further. Next, a transcriptional terminator sequence encoded by *rrnB *was PCR amplified using primers rrnB-*Kpn*I-fw (5'-TAAGGTACCCGGGGATCCTCTAGAGTCG-3') and rrnB-*Kpn*I-rv (5'-CGCGGTACCAAGAGTTTGTAGAAACGCAAA-3'), which both included *Kpn*I-site overhangs, and plasmid pSCrhaB1 [36] as a template. The 472-bp PCR fragment was digested with *Kpn*I and cloned into pBHR-MCS1. The correct orientation of the *rrnB *insert in the resulting plasmid pBHR1-MR was confirmed by PCR using primers rrnB-fw (5'-TCAGAAGTGAAACGCCGTAG-3') and cat1-rv (5'-ACGTGGCCAATATGGACAAC-3'). Next, a synthetic gene encoding a variant of the far-red fluorescent protein TurboFP635 (scientific name Katushka) was obtained from Source BioScience (formerly Geneservice). The variant *turboFP635 *sequence had been adapted to the codon bias of *B. pseudomallei *and was preceded by a *Spe*I site and followed by an *EcoR*V site. The 810-bp *turboFP635 *gene was cut from the cloning vector and cloned into *EcoR*V/*Spe*I restricted pBHR1-MR, resulting in plasmid pBHR1-RFP. Finally, a 443-bp fragment spanning the upstream region of the *groES *gene on chromosome I of *B. pseudomallei *strain K96243 (BPSL2698) was PCR amplified using primers groESprom-fw (5'-CTTGAGCTCGAACGTCGATTCGGACGCAT-3') and groESprom-rv (5'-GCGGACTAGTATTCACTCCTCTCTTTGATT-3'), which included *Sac*I and *Spe*I restriction sites, respectively. The PCR product was cloned into pBHR1-RFP via its *Sac*I/*Spe*I sites, resulting in plasmid pBHR1-groS-RFP (Km^R^, Cm^R^). For use in intracellular replication assays, the kanamycin resistance cassette of plasmid pBHR1 and the derivatives described had to be eliminated by the following method. Firstly, unmethylated pBHR1 plasmid DNA isolated from a *dcm*^-^/*dam*^- ^*E. coli *strain C2925 (New England Biolabs) was cut with *Stu*I/*PpuM*I, which resulted in a 1.2-kb fragment encompassing the kanamycin resistance cassette and a 4.1-kb plasmid backbone fragment. The 4.1-kb fragment was treated with T4 DNA polymerase (Promega) according to the manufacturer's recommendations and re-ligated overnight at 15°C resulting in plasmid pBHR4 (Cm^R^). Finally, a 1-kb fragment representing the *cat *gene of plasmid pBHR4 was replaced by a 3.2-kb fragment of plasmid pBHR1-groS-RFP, which encompassed the RFP gene linked to the *groES *promoter, the *rrnB *terminator and the *cat *gene, via *BstB*I restriction as described for the construction of pBHR-MCS1&2. This resulted in plasmid pBHR4-groS-RFP (Cm^R^).

### Macrophage uptake and intracellular survival assays

*Burkholderia *uptake and survival were quantified utilising a modified kanamycin protection assay. An overnight culture of bacteria was pelleted and resuspended at 1 × 10^6 ^cells/ml in Leibovitz L-15 medium supplemented with L-glutamine and L-Amino acids (Gibco). The bacterial suspensions were then added onto J774A.1 murine macrophages that had been seeded at 1 × 10^5 ^cells/ml in 24-well plates, thereby resulting in a multiplicity of infection (MOI) of 10:1. The monolayers were incubated at 37°C for 2 hrs to allow bacterial internalisation to occur. Cells were washed with PBS and L-15 medium containing 250 μg/ml kanamycin was added to suppress the growth of extracellular bacteria. At appropriate time points, cells were washed with warm PBS and lysed in 0.1% Triton X-100 in PBS for 5 mins. The lysis mixture was diluted and appropriate dilutions plated out on LB agar plates which were then incubated overnight at 37°C to allow bacteria to grow. All experiments were performed in triplicate with three technical replicates each.

### Cytotoxicity Assay (LDH assay)

Culture supernatants were harvested from infected J774A.1 macrophage monolayers at various time points as described above. The LDH assay was carried out using a CytoTox 96 Non-Radioactive Cytotoxicity Assay according to the manufacturer's protocol (Promega). Results were analysed using a Biorad Model 680 plate reader at OD 490 nm. Supernatants from uninfected macrophages were used as a control and the observed OD 490 nm readings were subtracted from the sample readings in order to correct for the background. All experiments were performed in triplicate with three technical replicates each.

### Multinucleated giant cell (MNGC) formation

J774A.1 macrophages were infected as already described. At appropriate time points, cells were washed with PBS and acid ethanol treated (5% acetic acid (v/v), 5% dH_2_O and 90% Ethanol (v/v)) for 30 mins at room temperature. Cells were thoroughly washed with PBS and stained with Giemsa solution (0.1% w/v) for 30 mins at room temperature. After washing with dH_2_O, cells were allowed to dry before being visualised under a light microscope. At least 10 fields per view at 10 × magnification were analysed for the percentage of MNGCs, where a cell was considered a MNGC if 3 or more nuclei were present.

### Confocal microscopy

J774A.1 macrophages grown on glass coverslips placed at the bottom of 24-well plates were infected with *Burkholderia *strains transformed with plasmid pBHR4-groS-RFP at an MOI of 10 as already described. At appropriate time points, cells were washed three times with warm PBS and fixed with 4% paraformaldehyde for 15 mins at room temperature. Cells were washed three times with PBS for 5 mins each before permealising the cells with 0.1% Triton X-100 in PBS for 30 mins at room temperature. After three more washes in PBS, filamentous actin was stained with 1 μg/ml FITC-phalloidin conjugate solution (Sigma) in PBS for 1 hr at room temperature. After several washes in PBS to remove unbound phalloidin conjugate, coverslips were mounted onto microscopy slides using Vectashield mounting medium containing DAPI (Vector Laboratories). Samples were analysed using a ZEISS LSM510 Meta confocal-laser scanning microscope.

### *Galleria mellonella *killing assays

Wax moth larvae (*Galleria mellonella*) were purchased from Livefood UK Ltd (Rooks Bridge, Somerset, UK) and were maintained on wood chips in the dark at 15°C until used. Bacteria from overnight cultures were adjusted to a known concentration in PBS and a Hamilton syringe was used to inject 10 μl aliquots of this suspension into *G. mellonella *larvae. Injections were performed into the haemocoel of 10 larvae per bacterial strain via the foremost left proleg. Control larvae were either injected with 10 μl of PBS in order to measure any potential lethal effects of the injection process, or not injected to measure the effects of the incubation procedure. After injection, larvae were incubated statically at 37°C inside petridishes and the number of dead larvae was scored periodically. Larvae were considered dead when they displayed no movement in response to gentle prodding with a pipette tip. To determine intracellular bacterial numbers, infected larvae were placed on ice for 20 mins before the bottom 2 mm of each larva was aseptically removed and the haemocoel was drained into a sterile 1.5 ml microcentrifuge tube on ice. This was then serially diluted in LB medium and appropriate dilutions were plated out onto LB agar plates supplemented with gentamicin, which were incubated overnight at 37°C to allow bacteria to grow. All experiments were carried out in triplicate.

### Statistical analysis

Differences between mean values were tested for significance by performing unpaired, two-tailed Student's t-tests using the GraphPad Prism software version 5.01 (GraphPad Software, San Diego California USA).

## Authors' contributions

MEW contributed to the experimental design, carried out the experiments and drafted the manuscript. CMM has constructed the fluorescent reporter plasmid and coordinated and edited the manuscript. RWT participated in study design and coordination and contributed to the manuscript. SLM conceived and coordinated the experimental design of the study and contributed to the manuscript. All authors read and approved the final manuscript.
